# Information-theoretic analysis of Hierarchical Temporal Memory-Spatial Pooler algorithm with a new upper bound for the standard information bottleneck method

**DOI:** 10.3389/fncom.2023.1140782

**Published:** 2023-06-07

**Authors:** Shiva Sanati, Modjtaba Rouhani, Ghosheh Abed Hodtani

**Affiliations:** ^1^Department of Computer Engineering, Ferdowsi University of Mashhad, Mashhad, Iran; ^2^Department of Electrical Engineering, Ferdowsi University of Mashhad, Mashhad, Iran

**Keywords:** Spatial Pooler (SP), Hierarchical Temporal Memory (HTM), sparsity, standard information bottleneck (IB), modified-information bottleneck (modified-IB), Fisher information matrix (FIM), Cramer-Rao lower bound (CRLB)

## Abstract

Hierarchical Temporal Memory (HTM) is an unsupervised algorithm in machine learning. It models several fundamental neocortical computational principles. Spatial Pooler (SP) is one of the main components of the HTM, which continuously encodes streams of binary input from various layers and regions into sparse distributed representations. In this paper, the goal is to evaluate the sparsification in the SP algorithm from the perspective of information theory by the information bottleneck (IB), Cramer-Rao lower bound, and Fisher information matrix. This paper makes two main contributions. First, we introduce a new upper bound for the standard information bottleneck relation, which we refer to as modified-IB in this paper. This measure is used to evaluate the performance of the SP algorithm in different sparsity levels and various amounts of noise. The MNIST, Fashion-MNIST and NYC-Taxi datasets were fed to the SP algorithm separately. The SP algorithm with learning was found to be resistant to noise. Adding up to 40% noise to the input resulted in no discernible change in the output. Using the probabilistic mapping method and Hidden Markov Model, the sparse SP output representation was reconstructed in the input space. In the modified-IB relation, it is numerically calculated that a lower noise level and a higher sparsity level in the SP algorithm lead to a more effective reconstruction and SP with 2% sparsity produces the best results. Our second contribution is to prove mathematically that more sparsity leads to better performance of the SP algorithm. The data distribution was considered the Cauchy distribution, and the Cramer–Rao lower bound was analyzed to estimate SP’s output at different sparsity levels.

## 1. Introduction

Hierarchical Temporal Memory (HTM) is an unsupervised learning algorithm and a unique artificial intelligence method inspired by the neocortex (Hawkins et al., 2016). The neocortex plays an important role in the human cerebral cortex, accounting for about half of the brain’s volume. It is responsible for behavioral and emotional responses and the greatest cognitive functions (Ghazanfar and Schroeder, 2006; Wang, 2022). The neocortex has a hierarchical and homogeneous structure in which higher parts learn general features and lower parts process stimuli (Clark et al., 2002; O’Reilly and Norman, 2002; Friston and Buzsáki, 2016). The neocortex consists of neurons, synapses, and segments (Menzel and Giurfa, 2001). Through synapses and segments, neurons can communicate with one another (Marois and Ivanoff, 2005). Essentially, two types of horizontal and vertical connections transmit information to the cell through the synapse. Horizontal connections represent context inputs, and vertical connections represent feedback and feedforward information (Barack and Krakauer, 2021). HTM is a theoretical model that resembles the neocortex in many respects; for example, it can memorize sequences and then recall them. With the help of a tree-shaped hierarchy neural network, The HTM algorithm extends and combines techniques used in bayesian networks, spatial and temporal clustering algorithms, and sparse distributed memory. It is a new model of the deep learning process, which is a highly efficient technique in artificial intelligence algorithms. HTM is an online learning method that does not require multiple training epochs. It is a one-shot learning process because almost all the necessary synaptic connections are formed in the first learning round. This algorithm is able to predict and recognize sequences with such robustness without suffering from the usual limitations of conventional neural networks that hinder their training. HTM is a predictive framework, so upon the model receiving each new input, it tries to predict the next events of the world. The HTM algorithm is not only used to detect the next value in a sequence but also to detect anomalies in a sequence. There are four components in HTM: SDR Encoder, Spatial Pooler (SP), Temporal Memory (TM), and Classifier. SP is one of the main components of the HTM, which continuously encodes streams of binary sensory input from various layers and regions within the neocortex into sparse distributed representations (SDR) (Cui et al., 2017). So the information is processed sparsely and encoded inside the HTM neurons as in biological neuronal networks (Finelli et al., 2008).

In Spatial Pooler, similar spatial patterns are grouped into highly sparse output representations of cortical mini-columns (Kaas, 2012; Ahmad and Scheinkman, 2019). In SP, there are two main tasks: the first task is to produce similar sparse outputs for similar inputs. The second critical task is to ensure that the output sparsity is fixed regardless of the number of bits in the binary input. Like normalization in other neural networks, these properties act as constraints on the behavior of neurons, which facilitates the training process (Hawkins et al., 2011). The SP algorithm is based on sparse coding techniques. According to the sparse coding theory, sparse activations in the brain’s sensory cortex reduce brain energy consumption while maintaining most of the information (Foldiak, 2003; McClelland and Bayne, 2016). In sparse coding, the cost function is optimized to combine a low reconstruction error with a high sparsity (Olshausen and Field, 1996). The receptive fields produced by sparse coding when applied to natural images are similar to those of brain V1 neurons (visual area); consequently, the sparse coding framework appears responsible for explaining early sensory neurons’ functionality (Lee et al., 2006; Paiton et al., 2020). The sparse representation is noise-resistant, and it is suitable for face recognition (Wei et al., 2022), speech recognition (Kwek et al., 2022), and image reconstruction (Deeba et al., 2020).

The sparse coding aims to form associative memory with minimal crosstalk, reduce power consumption, and prevent information loss (Hu and Zeng, 2022). So, in addition to the usefulness of sparsity in the HTM-SP algorithm, the concept of sparsity is helpful in various fields, such as image processing (Peng et al., 2018), medical imaging (Li et al., 2021), machine learning algorithms (Li, 2013), dictionary learning (Wang et al., 2019), denoising (Zhou et al., 2022), sampling theory (Nagahara and Yamamoto, 2022), and signal recovery (Abiantun et al., 2019). In the sparse representation models, a small number of coefficients contain a significant amount of energy (Ravishankar and Bresler, 2012). The SP algorithm has a fixed-sparsity representation of the input (Ahmad and Hawkins, 2016). A fixed level of sparsity in presynaptic inputs results in reliable and robust recognition of presynaptic activation patterns (Olshausen and Field, 2004). In the case of highly variable sparsity, it is difficult to detect input patterns with low activation density. On the other hand, input patterns with high activation density cause action potentials in downstream neurons. Therefore, false negative errors will occur in the case of low-density patterns, and false positive errors will occur in the case of high-density patterns. In general, it is desirable to have a fixed sparsity since it ensures that all input patterns can be detected equally. The fixed sparsity is approximately 2%, it means that only 2% of columns in the SP algorithm are activated (Hawkins et al., 2016).

### 1.1. Our contributions

This paper analyzes the sparsification in the HTM-SP algorithm from an information theory perspective. So, we applied the IB relation to investigate the accuracy of SP output reconstruction at different sparsity levels and various amounts of noise for the first time. Moreover, we proposed a new MNIST relation, which was employed to resolve the reconstruction problem. Then the Fisher information matrix and Cramer-Rao lower bound are used to prove mathematically that more sparsity leads to better performance of the HTM-SP algorithm. The data distribution is considered the Cauchy distribution, although similar analyses could have been conducted with other distributions. Here, the modified-IB is introduced as an upper bound for IB. Furthermore, its applicability is tested on the sparsity-noise impact of SP algorithm by using both standard and modified-IB. However, the application of the modified-IB is by no means limited to the HTM or its SP algorithm. In fact, the modified-IB can be used in any other study, replacing the standard IB.

### 1.2. Paper organization

The structure of this paper is arranged as follows. Section “2 Preliminaries” reviews all the preliminary details to improve the paper’s readability. In section “3 Our work,” our work is explained. Numerical experiments and simulations of the proposed modified-IB method are in section “4 Numerical Results related to our new IB relation.” Finally, in section “5 Conclusion,” the paper is concluded.

## 2. Preliminaries

The purpose of this section is to explain some of the scientific terms used in the rest of the paper, including the information bottleneck^1^ relation, Fisher information Matrix^2^, and Cramer-Rao lower bounds^3^.

### 2.1. Information bottleneck

**FIGURE 1:**
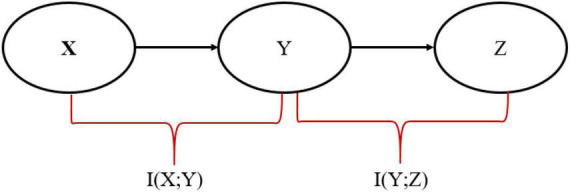
Representation of Markov Chain in the information bottleneck (IB) framework.

The Information bottleneck (IB) method is introduced by Tishby et al. (2000) and is used to find a maximally compressed representation in the *X* → *Y* → *Z* Markov chain that transmits information from input random variable X to output random variable Z through a compressed representation Y to preserve as much relevant information as possible, as shown in Figure 1.

The objective of the IB is to find the optimal representation Y, which involves minimizing the following Lagrangian cost function:


(1) $$L_{I\,B}=I\,\left(X;Y\right)-\beta \,I\,\left(Y;Z\right)$$

Where *I*(_; _) is the mutual information. *I*(*X*; *Y*) represents the compression or pruning term that discards irrelevant information by minimizing the mutual information between Y and source X and *I*(*Y*; *Z*) preserving relevant information to ensure Y predicts Z by maximizing the mutual information between Y and Z. The multiplier β ≥ 0 is a hyperparameter that controls the trade-off between these two terms (Alemi et al., 2016). A small β generally indicates more compression, whereas a large β indicates that more relevant information is being maintained.

### 2.2. Fisher information and Cramer–Rao lower bound definition

The Fisher information matrix (FIM) measures the amount of information the data can provide about the unknown parameter in an estimation problem (Stein et al., 2014). In order to quantify the Fisher information (FI) based on log-likelihoods, the following definitions are presented:


(2) $$I\,\left(\theta \right)=E_{\theta }\,\left\{{\left[l^{\prime }\,\left(x;\theta \right)\right]}^{2}\right\}=\int {\left[l^{\prime }\,\left(x;\theta \right)\right]}^{2}\,f\,\left(x;\theta \right)\,dx$$

(3) $$I\,\left(\theta \right)=-E_{\theta }\,\left\{\left[l^{\prime \prime }\,\left(x;\theta \right)\right]\right\}=-\int \left[\frac{\partial^{2}}{\partial \theta^{2}}\,\log f\,\left(x;\theta \right)\right]\,f\,\left(x;\theta \right)\,dx$$

In these equations, *f*(*x*; θ) is the probability distribution function of a random variable X, where X depends on the parameter θ ∈ Θ and *l*(*x*; θ) denotes the log-likelihood function.

The Fisher information of the probability family is a symmetric and positive semi-definite matrix valued function, where the *ij*th entry is as follows:


(4) $$I_{i,j}\,\left(\theta \right)=E_{\theta }\,\left[\left(\frac{\partial }{\partial \theta_{i}}\,\log p_{\theta }\,\left(X\right)\right)\,\left(\frac{\partial }{\partial \theta_{j}}\,\log p_{\theta }\,\left(X\right)\right)\right]$$

The Cramer-Rao inequality, which is the right-hand side expression in (5), explains the relationship between FIM and error variance in the following manner. It is almost a direct result of a famous mathematics inequality known as the Cauchy-Schwartz inequality (Hoffmann and Kunze, 1971).


(5) $$v\,a\,r\,\left[\hat{\theta }\right]\geq \frac{1}{n\,I\,\left(\theta \right)}$$

In this case, n represents the sample vector’s size, *I*(θ) represents the Fisher Information Matrix (FIM), and $\hat{\theta }$ represents the unbiased estimator of θ.

## 3. Our work

### 3.1. A new upper bound for IB relation on the SP algorithm

The learning aim is to find a function that minimizes the uncertainty of the output given the input while avoiding irrelevant information as much as possible. Tishby et al. (2000) introduced this viewpoint as the Information Bottleneck (IB), a fundamental concept in information theory.

This paper aims to apply the IB relation, for the first time, to analyze the effect of sparsity and noise on data reconstruction in the HTM-Spatial pooler algorithm and propose a modified-IB relation.

The IB method has an extraordinary application in various fields of machine learning and related domains (Riguzzi and Di Mauro, 2012; Goldfeld and Polyanskiy, 2020; Zuo et al., 2021; Musat and Andonie, 2022). It also applies to other areas, such as neuroscience (Schneidman et al., 2001; Buddha et al., 2013; Tucker et al., 2022). Tishby and Zaslavsky (2015) used the IB method to evaluate the deep neural networks’ performance and determine the reasons for their success. Despite the impressive successes of deep neural networks, they have been criticized for the lack of sufficient information from inside the network and for being unable to understand the internal structure and optimization process in recent years. The IB method has almost solved this problem and opened the deep learning black box. The experimental evidence in Shwartz-Ziv and Tishby (2017) indicated that deep neural networks implicitly solve the information bottleneck optimization problem, i.e., compress the input while preserving the associated information related to the output. The IB principle has been applied to complex and high-dimensional data in numerous studies, including improving and analyzing the learning of deep neural networks (Gu et al., 2020; Raj et al., 2020; Li and Liu, 2021; Vera et al., 2022), learning disentangled and invariant representations (Achille and Soatto, 2018), and enhancing robustness against adversarial attacks (Fischer, 2020). Scientists are still interested in this topic as it is still an open issue.

**FIGURE 2:**
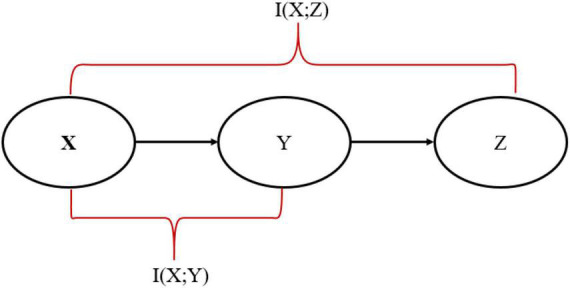
Representation of Markov Chain in the modified-information bottleneck (IB) framework.

**FIGURE 3:**
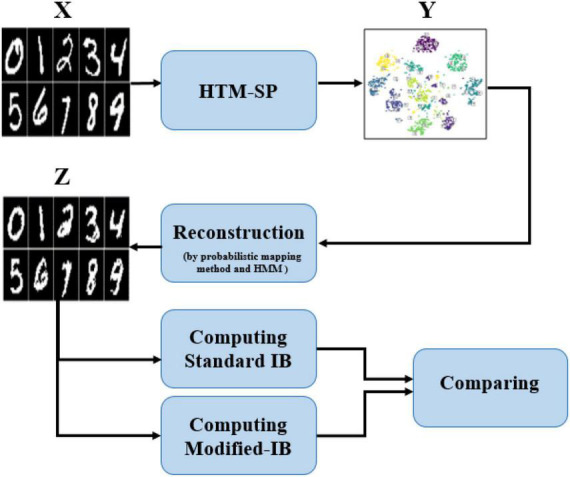
A block diagram of the proposed method. The Hierarchical Temporal Memory (HTM)-Spatial Pooler (SP) output at various sparsity levels and different amounts of noise are reconstructed by the probabilistic mapping method and Hidden Markov Model (HMM) algorithm; Afterward, the standard information bottleneck (IB) and modified IB are computed and compared with each other.

In this paper, the SP algorithm was fed with 60,000 MNIST data as a training set and produced the sparse representation. A probabilistic mapping method and Hidden Markov Model (HMM) were used to reconstruct the sparse SP output representation in the input space (Mnatzaganian et al., 2017). Although some information has been lost during the reconstruction process, it is unquestionable that the reconstructed data is similar to the original data. Using the standard IB relation (described in equation 6), we can accurately assess the similarity between input data and the data reconstructed by the SP algorithm at various sparsity levels and various amounts of noise. Therefore, we analyze how sparsity and noise affect reconstruction accuracy using the standard IB. It is the first time someone has calculated the standard IB relation for the SP algorithm and quantitatively compared the reconstructed data with the original data. Furthermore, we proposed a modified- IB relation (Figure 2), which was applied to measure the similarity in the SP’s reconstruction problem. Then we compare these two measures, as shown in Figure 3.


(6) $$L_{I\,B}=I\,\left(X;Y\right)-\beta_{1}\,I\,\left(Y;Z\right)$$

(7) $$L_{M-I\,B}=I\,\left(X;Y\right)-\beta_{2}\,I\,\left(X;Z\right)$$

The mathematical comparison of *L*_*IB*_ and *L*_*M–IB*_ (assuming β_1_ = β_2_) easily demonstrates that *L*_*M–IB*_ is greater than *L*_*IB*_. Because in the *X* → *Y* → *Z* Markov chain, it is obvious that as the distance between two nodes increases, more information is lost along the way:


(8) I⁢(X;Z)≤I⁢(Y;Z)

So,


(9) $$L_{M-I\,B}\geq L_{I\,B}$$

It was stated in section “2.1 Information bottleneck” that *L*_*IB*_ represents the information bottleneck, which should be minimized. As a result, we have proved that *L*_*M*−*IB*_ ≥ *L*_*IB*_. If we decrease *L*_*M–IB*_, *L*_*IB*_ must also decrease because it is less than *L*_*M*−*IB*_.

So, min(*L*_*M–IB*_) ≥ min(*L*_*IB*_). Therefore, we demonstrate *L*_*M–IB*_ is an upper bound of the information bottleneck method, and its minimization incorporates additional information. *L*_*M–IB*_ minimization also includes the information derived from the information bottleneck method.

### 3.2. The corresponding comparisons on the SP algorithm

A lower bound on variance is a handy feature of any unbiased estimator. Using this lower bound, selecting the most appropriate estimator based on possible minimal variance is possible (Tune, 2012; Huang et al., 2020; Khorasani et al., 2020). The Cramer-Rao Lower Bound (CRLB) is the most popular lower bounds in the literature due to its attractiveness and ease of evaluation (Rao et al., 1973; D’Amico et al., 2022). As stated in the Cramer-Rao inequality (Dogandzic and Nehorai, 2001), the diagonal terms of the inverse of the Fisher information Matrix (FIM) (assuming it exists) represent asymptotic lower bounds on any unbiased estimator’s variance. The Fisher information matrix measures the amount of information the data can provide about the unknown parameter in an estimation problem (Stein et al., 2014). A parameter can be more accurately estimated when the data contains more information about the parameter. Information amount is determined by considering how the likelihood of observing the acquired data changes with the parameter value. In the absence of a significant change in the likelihood of the data with respect to the parameter value, the data contains very little information about the parameter. The Cramer–Rao lower bound can be used as a benchmark to determine the accuracy of a method. When the estimator’s variance is equal to the CRLB, it is considered the most efficient estimator. The purpose of this section is to demonstrate that CRLB will be decreased as a consequence of data sparsification. The claims are proved by using the Cauchy distribution. We first examine the sparsity variation of output caused by the filter in the SP algorithm and then explore three scenarios (as described below) in which the CRLB of the estimation error is calculated to examine the effect of SP’s output sparsity on the estimation error.

• Compute CRLB when SP’s output is not sparse.
• Compute CRLB when SP’s output has the maximum Sparsity.
• Compute CRLB when adding noise to the SP’s output.

Let’s consider the first scenario where the input data (X) follows the Cauchy distribution, so *x*_θ_ ∼ *C*(*x*_0_, γ) and θ = (*x*_0_, γ)^*T*^; therefore, multiple parameters must be estimated. The first step in determining CRLB is defining a Fisher information matrix for the two main parameters of Cauchy distribution (*x*_0_ and γ). A log-likelihood function *l*(*x*; θ) based on the Cauchy distribution is written as follows.


(10) $$f\,\left(x;\theta \right)=f\,\left(x;x_{0},\gamma \right)=\frac{1}{\pi \,\gamma \,\left[1+{\left(\frac{x-x_{0}}{\gamma }\right)}^{2}\right]}=$$

$$\frac{\gamma }{\pi \,\left(\gamma^{2}+{\left(x-x_{0}\right)}^{2}\right)}$$

Where *x*_0_ indicates the location parameter of the peak and shifts the graph along the x-axis, and γ indicates the scale parameter of the graph, which can be either shorter or taller. And then


(11) $$l\,\left(x;\theta \right)=\ln f\,\left(x;\theta \right)=\ln\left(\gamma \right)-\ln\left(\pi \right)-\ln\left(\gamma^{2}+{\left(x-x_{0}\right)}^{2}\right)$$

The Fisher information matrix can be determined by computing the first and second derivatives of ln *f*(*x*; θ):


(12) $$\frac{\partial l\,\left(x;\theta \right)}{\partial \theta }={\left(\frac{\partial l\,\left(x;\theta \right)}{\partial x_{0}},\frac{\partial l\,\left(x;\theta \right)}{\partial \gamma }\right)}^{T}=$$

$$\left(\frac{2\,\left(x-x_{0}\right)}{\gamma^{2}+{\left(x-x_{0}\right)}^{2}},\frac{1}{\gamma }-\frac{2\,\gamma }{\gamma^{2}+{\left(x-x_{0}\right)}^{2}}\right)$$

And


(13) $$\frac{\partial^{2}l\,\left(x;\theta \right)}{\partial \theta^{2}}=\left[\begin{array}{cc} \frac{\partial^{2}l\,\left(x;\theta \right)}{\partial x_{0}^{2}}\,\frac{\partial^{2}l\,\left(x;\theta \right)}{\partial x_{0}\,\partial \gamma } & \\ \frac{\partial^{2}l\,\left(x;\theta \right)}{\partial \gamma \,\partial x_{0}}\,\frac{\partial^{2}l\,\left(x;\theta \right)}{\partial \gamma^{2}} & \end{array}\right]=$$

$$\left[\begin{array}{ccc} \frac{-4\gamma^{2}+2[\gamma^{2}+{\left(x-x_{0}\right)}^{2}]}{{\left[\gamma^{2}+{\left(x-x_{0}\right)}^{2}\right]}^{2}} & & \frac{-4\gamma (x-x_{0})}{{\left[\gamma^{2}+{\left(x-x_{0}\right)}^{2}\right]}^{2}}\\ \frac{-4\gamma (x-x_{0})}{{\left[\gamma^{2}+{\left(x-x_{0}\right)}^{2}\right]}^{2}} & & \frac{-1}{\gamma^{2}}-\frac{2}{\gamma^{2}+{\left(x-x_{0}\right)}^{2}}+\frac{4\gamma^{2}}{{\left[\gamma^{2}+{\left(x-x_{0}\right)}^{2}\right]}^{2}} \end{array}\right]$$

Equation (14) provides Fisher’s information matrix, and the calculations related to this part are contained in the Supplementary Appendix 1.


(14) $$I(\theta )=-E_{\theta }[\frac{\partial^{2}l(x;\theta )}{\partial \theta^{2}}]=\left[\begin{array}{cc} \frac{1}{2\gamma^{2}} & 0\\ 0 & \frac{1}{2\gamma^{2}} \end{array}\right]$$

As a result, *x*_0_’s Fisher information contained in the random variable X is equivalent to $I_{11}\,\left(\theta \right)=\frac{1}{2\,\gamma^{2}}$, and γ’s Fisher information is equal to $I_{22}\,\left(\theta \right)=\frac{1}{2\gamma {}^{2}}$.

So, according to their general definition, the Cramer-Rao lower bound for the estimation error for both parameters *x*_0_ and γ is *CRLB* = *I*(θ)^−1^.


(15) $$C\,B\,R\,L\,B_{x_{0}}=I_{11}^{-1}\,\left(\theta \right)=2\,\gamma^{2}$$

(16) $$C\,B\,R\,L\,B_{\gamma }=I_{22}^{-1}\,\left(\theta \right)=2\,\gamma^{2}$$

This article examines the effect of Sparsity on the error bound of SP’s output reconstruction. For this purpose, we explore an ideal state of Sparsity (the maximum Sparsity) in which all the information is contained in one SP’s output element, and all the rest are zero after the sparsification process, *x* = [*x*_*sparse*_] so *x*_0_ = *x*_*sparse*_ and γ = 0.

By substituting γ = 0 in the Fisher information matrix in (14), the following result in (17) is obtained:


(17) $$I_{11}\,\left(\theta \right)=I_{22}\,\left(\theta \right)=\infty$$

Therefore, the Cramer-Rao lower bound for these two parameters is equal to zero, which means that the SP’s output reconstruction error is zero when we have the SP with maximum Sparsity.


(18) $$C\,B\,R\,L\,B_{x_{0}}=C\,B\,R\,L\,B_{\gamma }=0$$

Tests were conducted with other distributions, including Normal, Poisson, Exponential, and Bernoulli. Once again, the results indicated that the CRLB is zero when the Sparsity of the SP’s output is maximum.

Next, we assume that the SP’s output has decreased its Sparsity. For example, we decrease SP’s output sparsity by adding a constant value λ to all of its elements. The aim is to investigate the relation between the Cramer-Rao lower bound and decreasing the SP’s output sparsity.

Let’s assume that the input data (X) follows the Cauchy distribution, so *x*_θ_ ∼ *C*(*x*_0_,γ). In this case, the parameters of the new vector (X + λ) will change as follows:


(19) $$x_{\lambda }=x_{0}\,\left(\lambda +X_{\theta }\right)=\lambda +x_{0}\,\left(X_{\theta }\right)=\lambda +x_{0}$$

(20) $$\gamma_{\lambda }=\gamma \,\left(\lambda +X_{\theta }\right)=\gamma \,\left(X_{\theta }\right)=\gamma$$

To incorporate these changes, the second derivative of the log-likelihood function in equation (13) is rewritten as follows:


(21) $$\frac{\partial^{2}l\,\left(x;\theta \right)}{\partial \theta^{2}}=$$

$$\left[\begin{array}{ccc} \frac{-4\gamma^{2}+2[\gamma^{2}+{\left(x-x_{0}-\lambda \right)}^{2}]}{{\left[\gamma^{2}+{\left(x-x_{0}-\lambda \right)}^{2}\right]}^{2}} & & \frac{-4\gamma (x-x_{0}-\lambda )}{{\left[\gamma^{2}+{\left(x-x_{0}-\lambda \right)}^{2}\right]}^{2}}\\ \frac{-4\gamma (x-x_{0}-\lambda )}{{\left[\gamma^{2}+{\left(x-x_{0}-\lambda \right)}^{2}\right]}^{2}} & & \frac{-1}{\gamma^{2}}-\frac{2}{\gamma^{2}+{\left(x-x_{0}-\lambda \right)}^{2}}+\frac{4\gamma^{2}}{{\left[\gamma^{2}+{\left(x-x_{0}-\lambda \right)}^{2}\right]}^{2}} \end{array}\right]$$

Therefore, The Fisher information matrix can be expressed as follows:


(22) $$I(\theta )=-E_{\theta }[\frac{\partial^{2}l(x;\theta )}{\partial \theta^{2}}]=\left[\begin{array}{cc} \frac{1}{2\gamma^{2}} & 0\\ 0 & \frac{1}{2\gamma^{2}} \end{array}\right]$$

The CRLB, which is the inverse matrix of Fisher information, can be expressed as follows:


(23) $$C\,B\,R\,L\,B_{x_{0}}=I_{11}^{-1}\,\left(\theta \right)=2\,\gamma^{2}$$

(24) $$C\,B\,R\,L\,B_{\gamma }=I_{22}^{-1}\,\left(\theta \right)=2\,\gamma^{2}$$

This analysis allows us to compare the CRLB in equations (23) and (24) to the CRLBs calculated for the SP’s output before adding constant λ value, equations (15) and (16). Explicitly, it is shown that a decrease in sparsity does not change the Cramer-Rao lower bound in the Cauchy distribution. It should be noted that, in this particular test, if the data followed a Gaussian distribution, the decrease in sparsity would increase the Cramer-Rao lower bound (Khorasani et al., 2020). Consequently, a Gaussian distribution is considered the worst-case scenario in estimating unknown parameters. As stated before, these experiments can be performed with other distributions, including Gaussian, Pareto, Poisson, Exponential, and Bernoulli. Table 1 summarizes the properties of these five distributions, as well as the Fisher information matrix and the Cramer-Rao lower bound.

**TABLE 1:** Some other distributions that can be used in our experiments.

Distribution	PDF/PMF	Parameter	Fisher information	Cramer-Rao lower bound
Gaussian	$\frac{1}{\delta \,\sqrt{2\,\pi }}\,\exp-\frac{\left(x-\mu \right)}{2\,\delta^{2}}$	μ, δ^2^	$\left(\begin{array}{cc} \frac{1}{\delta^{2}} & 0\\ 0 & \frac{1}{2\,\delta^{4}} \end{array}\right)$	$\begin{array}{l} CRLB_{\text{μ}}={\text{δ}}^{2}\\ CRLB_{{\text{δ}}^{2}}=2{\text{δ}}^{4} \end{array}$
Pareto	$\frac{\alpha \,x_{m}^{\alpha }}{x^{\alpha +1}}$	*x*_*m*_ > 0 α > 0	$\left(\begin{array}{cc} \frac{\alpha^{2}}{x_{m}^{2}} & 0\\ 0 & \frac{1}{\alpha^{2}} \end{array}\right)$	$\begin{array}{c} C\,R\,L\,B_{x_{m}}=\frac{x_{m}^{2}}{\alpha^{2}}\\ C\,R\,L\,B_{\alpha }=\alpha^{2} \end{array}$
Exponential	λ *exp*−λ*x*	λ > 0	$\frac{1}{\lambda^{2}}$	λ^2^
Poisson	$\frac{\lambda^{k}\,\exp-\lambda }{k!}$	λ ∈ (0,∞)	$\frac{1}{\lambda }$	λ
Bernoulli	{q=1−pif k=0pif k=1	0 ≤ *p* ≤ 1 *q* = 1 − *p*	$\frac{1}{p\,q}$	*pq*

## 4. Numerical results related to our new IB relation

This section examines the effect of different sparsity levels and various amounts of noise on the SP algorithm’s output reconstruction. The SP algorithm was fed with 60,000 MNIST data as a training set and produced the sparse representation. We add 0, 10, 20, 30, and 40% noise values to the input of the SP algorithm for each SP’s sparsity (Column-activation = 2, 10, 20, 30, and 40%), and the output is obtained. A probabilistic mapping method and Hidden Markov Model were used to reconstruct the sparse SP output representation in the input space (Mnatzaganian et al., 2017). The SP algorithm was simulated using the mHTM^4^ implementation. To examine the accuracy of the SP output reconstruction, we used the standard IB relation and proposed a new upper bound for it. The results of comparing the modified-IB and standard IB at different sparsity levels of the SP algorithm are reported in Table 2.

**TABLE 2:** The comparison of the modified-information bottleneck (IB) and standard information bottleneck (IB) methods with different β values in the reconstruction of the Hierarchical Temporal Memory (HTM)-Spatial Pooler (SP) algorithm using different sparsity (column-activation parameter) on MNIST dataset.

	Method \ β	10^–2^	10^–1^	10^0^	10^1^
2% sparsity	Modified-IB	0.3247	0.3247	0.3242	0.3199
	Standard IB	0.3247	0.3244	0.3217	0.2947
10% sparsity	Modified-IB	0.3306	0.3306	0.3301	0.3256
	Standard IB	0.3306	0.3303	0.3277	0.3016
20% sparsity	Modified-IB	0.338	0.338	0.3378	0.3358
	Standard IB	0.338	0.3378	0.336	0.318
30% sparsity	Modified-IB	0.4013	0.4013	0.401	0.3988
	Standard IB	0.4013	0.4012	0.4001	0.3893
40% sparsity	Modified-IB	0.41	0.41	0.4097	0.407
	Standard IB	0.41	0.4099	0.409	0.4003

According to the above experiments, the most accurate reconstruction occurs when the sparsity of the SP algorithm is high (2% sparsity), whereas reducing the sparsity in the SP algorithm increases the reconstruction error. In this experiment, it can be seen that standard IB is always lower than or equal to modified-IB, which is in accordance with our previously stated fact that modified-IB is an upper bound for the standard IB.

Using the Fashion-MNIST and NYC-Taxi datasets, we repeated all of the experiments, and compared the Modified-IB and Standard IB methods with different β values in the reconstruction of the HTM-SP algorithm using different sparsity. As shown in Tables 3, 4, SP with 2% sparsity produces the best results and the Modified-IB is always greater than or equal to the standard IB in these two datasets, as expected.

**TABLE 3:** The comparison of the modified-information bottleneck (IB) and standard information bottleneck (IB) methods with different β values in the reconstruction of the Hierarchical Temporal Memory (HTM)-Spatial Pooler (SP) algorithm using different sparsity (column-activation parameter) on Fashion-MNIST dataset.

	Method \ β	10^–2^	10^–1^	10^0^	10^1^
2% sparsity	Modified-IB	0.3429	0.3429	0.3407	0.3209
	Standard IB	0.3429	0.3425	0.3389	0.3029
10% sparsity	Modified-IB	0.3608	0.3608	0.3586	0.3388
	Standard IB	0.3608	0.3604	0.3568	0.3208
20% sparsity	Modified-IB	0.3712	0.3712	0.3710	0.3692
	Standard IB	0.3712	0.3708	0.3672	0.3312
30% sparsity	Modified-IB	0.4355	0.4355	0.4351	0.4315
	Standard IB	0.4355	0.4353	0.4335	0.4155
40% sparsity	Modified-IB	0.4493	0.4493	0.4490	0.4463
	Standard IB	0.4493	0.4492	0.4482	0.4392

**TABLE 4:** The comparison of the modified-information bottleneck (IB) and standard information bottleneck (IB) methods with different β values in the reconstruction of the Hierarchical Temporal Memory (HTM)-Spatial Pooler (SP) algorithm using different sparsity (column-activation parameter) on NYC-Taxi dataset.

	Method \ β	10^–2^	10^–1^	10^0^	10^1^
2% sparsity	Modified-IB	0.1121	0.1121	0.1099	0.0901
	Standard IB	0.1121	0.1116	0.1071	0.0621
10% sparsity	Modified-IB	0.1488	0.1488	0.1461	0.1218
	Standard IB	0.1488	0.1483	0.1438	0.0988
20% sparsity	Modified-IB	0.1560	0.1560	0.1558	0.1540
	Standard IB	0.1560	0.1557	0.1530	0.1260
30% sparsity	Modified-IB	0.2284	0.2284	0.2281	0.2254
	Standard IB	0.2284	0.2282	0.2264	0.2084
40% sparsity	Modified-IB	0.2512	0.2512	0.2509	0.2482
	Standard IB	0.2512	0.2511	0.2502	0.2412

**FIGURE 4:**
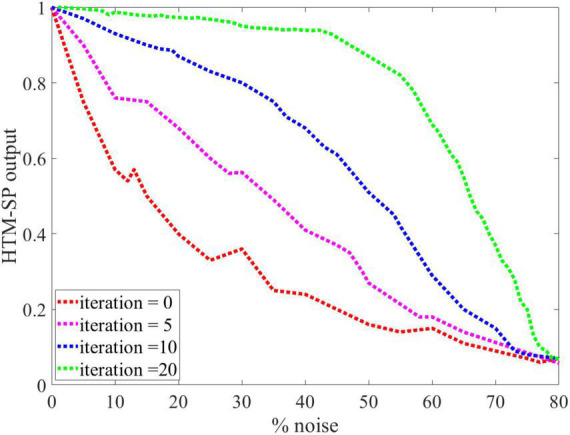
An analysis of the Spatial Pooler (SP) algorithm’s behavior against different levels of input noise during learning process by different iterations value is presented. The performance of the SP algorithm without learning (red line) is greatly affected by small amounts of noise. In contrast, even 40% noise does not significantly affect the output of the SP algorithm with learning (green line), after which the curve’s slope gradually changes.

Following this, we evaluate the noise robustness of the SP algorithm during the learning process by adding different amounts of noise to its input. The SP algorithm was trained using the noisy MNIST image as input for each noise level (between 0 and 75%) by randomly flipping active bits to inactive bits and inactive bits to active bits for the given percentage of the pixels. Twenty-one iterations are necessary for the learning process to ensure that the SP has a stable output representation. As shown in Figure 4, the SP algorithm with learning (green line) was more robust against noise than the SP algorithm without learning (red line). It was found that the learned outputs remained virtually unchanged (or with relatively small changes) after adding a significant amount of noise to the input, as it is almost smooth until 40% noise. Therefore, even adding up to 40% noise to the input resulted in no discernible change in the output of the SP algorithm with learning. The following experiments of this paper used the SP algorithm with learning due to its advantages over the SP without learning.

**FIGURE 5:**
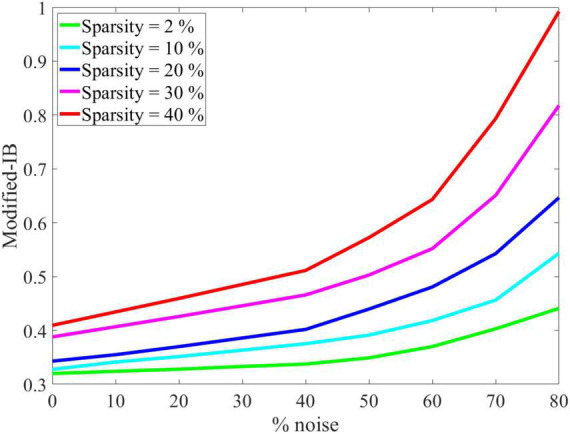
Noise effects on the modified-information bottleneck (IB) relation at β = 10 for different sparsity levels. With a higher sparsity level, the Spatial Pooler (SP) algorithm is more resistant to noise, and the modified-IB relation provides the best results.

As shown in Figure 5, the effect of SP’s sparsity and input noise on SP’s output reconstruction was quantified using the modified-IB relation. This experiment demonstrated that the original data performs better than the noisy data, and by increasing the sparsity of the SP algorithm, the noise entropy decreases. So, the addition of noise causes a higher reconstruction error. The IB curve is almost smooth, up to 40% noise, then changes dramatically. Therefore, adding up to 40% noise to the input results in no discernible change in the output of the SP algorithm. It is possible to measure the resistance to noise of the SP algorithm by the modified-IB relation (at different sparsity levels), and the result of Figure 5 is in accordance with the result of Figure 4.

**FIGURE 6:**
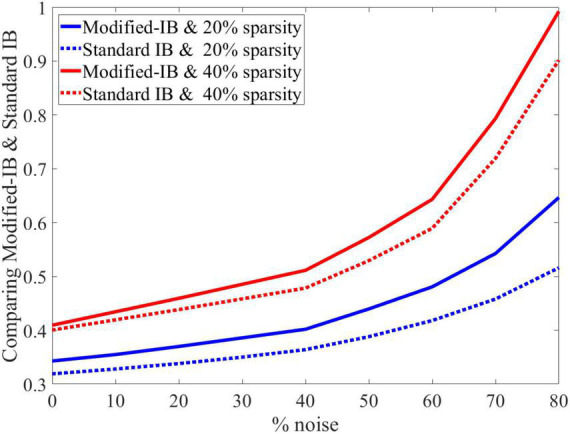
At different sparsity levels, standard information bottleneck (IB) is always lower than modified-IB.

As in the previous case, we concluded that the higher sparsity in the SP algorithm leads to a better reconstruction of the output, and standard IB is always lower than modified-IB (Figure 6).

**FIGURE 7:**
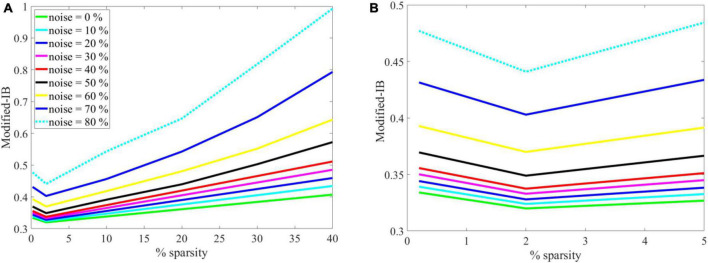
**(A)** The effects of sparsity on the modified-information bottleneck (IB) relation at β = 10 for different noise levels. With a lower noise level and sparsity = 2%, the modified-IB relation provides the best results. **(B)** The graph on the left has been zoomed in the range of 0–5 to gain more clarity and to show the sparsity = 2% is the most appropriate sparsity in the Spatial Pooler (SP) algorithm.

Again, the SP algorithm is quantitatively evaluated using the modified-IB relation at different sparsity levels and noise, as shown in (A) in Figure 7. For more clarity, in Figure 7 the graph (A) has been zoomed in the range of 0 to 5, as shown in (B). Accordingly, the (A) and (B) graphs in Figure 7 show some interesting results:

• If there is no noise (green line), modified-IB is smaller, and the SP algorithm is more accurate.
• If the sparsity level is low and the noise level is high, modified IB expresses a higher number indicating that the SP algorithm is inaccurate.
• The optimal situation is sparsity = 2% since modified-IB has the lowest value for all noises at this point.
• In the case of sparsity = 2%, the noises of 0 to 40% are almost adjacent, whereas the noises of 50–80% are far apart. Thus, we can conclude that adding up to 40% noise to the input results in no significant change in the output of the SP algorithm.

**FIGURE 8:**
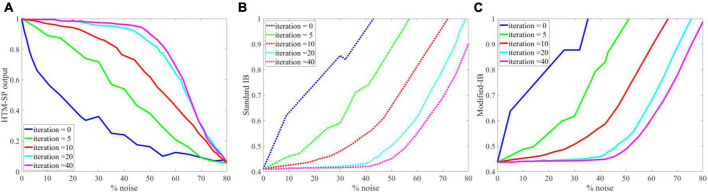
Analysis of the Spatial Pooler (SP) algorithm’s against different levels of input noise during learning process by different iterations value. **(A)** The change of the SP outputs is plotted as a function of the noise level (Cui et al., 2017). **(B)** Noise effects on the Standard information bottleneck (IB) relation at β = 10 for fixed sparsity 2% and in different iterations. **(C)** Noise effects on the Modified-IB relation at β = 10 for fixed sparsity 2% and in different iterations. In iteration 40, the SP algorithm is more resistant to noise, and the Standard IB relation provides the best results.

**FIGURE 9:**
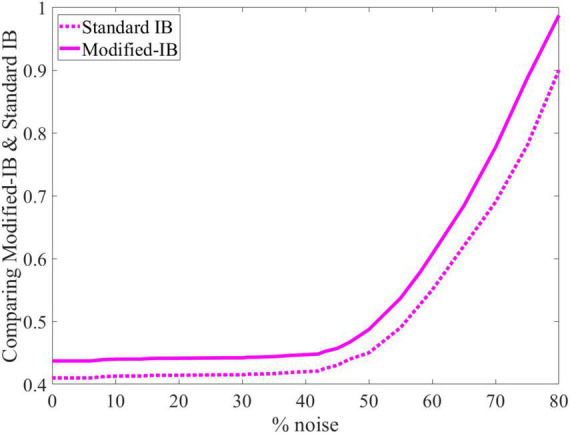
Modified-information bottleneck (IB) is an upper bound for the standard IB.

To further analysis the applicability of our method, we perform the same experience as in Cui et al. (2017) on random sparse inputs and tested the effect of noise on the SP algorithm in different iterations. In Figure 8A, presents the results of Figure 4 in reference (Cui et al., 2017). Figures 8B, C, are the results of application of IB and modified-IB in the same experience. In this figure an analysis of the SP algorithm’s behavior at different levels of input noise during the learning process with different iteration values is shown. The performance of the SP algorithm without learning (blue line) is greatly affected by small amounts of noise. In contrast, even 40% noise does not significantly affect the output of the SP algorithm with learning (Purple line), after which the curve’s slope gradually changes. As indicated in those figures, the results from reference (Cui et al., 2017) is in accordance with results from IB and Modified-IB. Figures 8B, C demonstrate that the SP algorithm preforms better when algorithm iterations are higher and the SP is in the learning phase (Purple line). In this case, even 40% noise does not significantly affect the SP algorithm output. In addition, Figure 9 shows that Modified-IB is an upper bound for Standard-IB, as we expected.

## 5. Conclusion

This paper aimed to evaluate the sparsification in the SP algorithm from the perspective of information theory as measured by the information bottleneck, Cramer-Rao lower bound, and Fisher information matrix. Two main contributions were made in this paper. First, we introduced a new upper bound for the standard information bottleneck relation. This measure has been used to evaluate the performance of the SP algorithm in different sparsity levels and various amounts of noise. The MNIST dataset was fed as input to the SP algorithm. The SP algorithm with learning was found to be resistant to noise. Adding up to 40% noise to the input resulted in no discernible change in the output. Using the probabilistic mapping method and Hidden Markov Model, the sparse SP output representation was reconstructed in the input space. The purpose was to assess the similarity between input data and the data reconstructed by the SP algorithm. SP with 2% sparsity produced the best results. The claims are numerically validated by the standard information bottleneck relation and its proposed new version (modified-IB). The results show that a lower amount of noise and a higher sparsity level in the SP algorithm improved reconstruction accuracy. This finding, which is based solely on information theory measures, is in coherence with empirical result. we have proved what was previously only an experimental observation. Our second contribution was to prove mathematically that more sparsity leads to better performance of the SP algorithm. So, we analyzed the relationship between the Cramer–Rao lower bound on the estimation of the SP’s output and the Sparsity of the SP’s output and also the relation of sparsity and adding noise to the SP’s output. Accordingly, this paper investigated the effects of varying the sparsity of the SP, followed by comparing the error bounds before and after sparsification. The data distribution was considered the Cauchy distribution, although similar analyses could have been conducted with other distributions, including Gaussian, Pareto, Poisson, Exponential, and Bernoulli. As a result of this research, it will be possible to reduce recovery errors during compression and transmission procedures.

## Data availability statement

Publicly available datasets were analyzed in this study, including the MNIST, Fashion-MNIST, and NYC-Taxi datasets. This data can be found here: http://yann.lecun.com/exdb/mnist/; https://github.com/zalandoresearch/fashion-mnist; and http://www.nyc.gov/html/tlc/html/about/trip_record_data.shtml.

## Author contributions

SS: conceptualization, methodology, software, validation, and writing—original draft. MR and GH: conceptualization, methodology, validation, supervision, project administration, and review and editing. All authors contributed to the article and approved the submitted version.
