# Supplementary material for: Information-theoretic analysis of Hierarchical Temporal Memory-Spatial Pooler algorithm with a new upper bound for the standard information bottleneck method
Source: Front Comput Neurosci. 2023 Jun 7;17:1140782. doi: 10.3389/fncom.2023.1140782 (PMC10282945; doi:10.3389/fncom.2023.1140782)
Supplement: Supplementary file 1 [file Data_Sheet_1.pdf]

Appendix 1

Fisher's information matrix calculations are presented here.

$$I(\theta) = -E_\theta[\frac{\partial^2 l(x;\theta)}{\partial\theta^2}] = -E_\theta \begin{bmatrix} \frac{-4\gamma^2 + 2[\gamma^2 + (x-x_0)^2]}{[\gamma^2 + (x-x_0)^2]^2} & \frac{-4\gamma(x-x_0)}{[\gamma^2 + (x-x_0)^2]^2} \\ \frac{-4\gamma(x-x_0)}{[\gamma^2 + (x-x_0)^2]^2} & \frac{-1}{\gamma^2} - \frac{2}{\gamma^2 + (x-x_0)^2} + \frac{4\gamma^2}{[\gamma^2 + (x-x_0)^2]^2} \end{bmatrix}$$

As a first step, $I_{11}(\theta)$ was calculated as follows:

$$I_{11}(\theta) = -E_\theta \left[\frac{-4\gamma^2 + 2[\gamma^2 + (x-x_0)^2]}{[\gamma^2 + (x-x_0)^2]^2} \right]$$

In order to quantify the $I_{11}(\theta)$ based on log-likelihoods, we use equation (2) as explained earlier in section 2-2 and it means as the expectation is continuous it is going to do integration. So,

$$I_{11}(\theta) = -\int_{-\infty}^{+\infty} \frac{-4\gamma^2 + 2[\gamma^2 + (x-x_0)^2]}{[\gamma^2 + (x-x_0)^2]^2} \cdot \frac{\gamma}{\pi(\gamma^2 + (x-x_0)^2)} dx$$

In addition, split it into two integrals as follow and try to solve them separately.

$$= \frac{4\gamma^3}{\pi} \int_{-\infty}^{+\infty} \frac{1}{[\gamma^2 + (x-x_0)^2]^3} dx - \frac{2\gamma}{\pi} \int_{-\infty}^{+\infty} \frac{1}{[\gamma^2 + (x-x_0)^2]^2} dx$$

Now, we do a variable change. After transformation $X = x - x_0$, we get the following result:

$$= \frac{4\gamma^3}{\pi} \underbrace{\int_{-\infty}^{+\infty} \frac{1}{[\gamma^2 + X^2]^3} dX}_{A1} - \frac{2\gamma}{\pi} \underbrace{\int_{-\infty}^{+\infty} \frac{1}{[\gamma^2 + X^2]^2} dX}_{A2}$$

Consequently, we needed to calculate relations (A1) and (A2), which are also given in the continuation of their computation, so the $I_{11}(\theta)$ result is as follows:

$$= \frac{4\gamma^3}{\pi} * \frac{1}{\gamma^5} * \frac{3\pi}{8} - \frac{2\gamma}{\pi} * \frac{1}{\gamma^3} * \frac{\pi}{2} = \frac{1}{2\gamma^2}$$

The calculations related to A1 and A2 are given below. First A1 is calculated:

$$\underbrace{\int_{-\infty}^{+\infty} \frac{1}{[\gamma^2 + X^2]^3} dX}_{A1} \qquad \text{(A1)}$$

The variable change is used. Let $x = \frac{X}{\gamma}$, So

$$\underbrace{\int_{-\infty}^{+\infty} \frac{1}{[\gamma^2 + X^2]^3} dX}_{A1} = \frac{1}{\gamma^5} \int_{-\infty}^{+\infty} \frac{1}{(1+x^2)^3} dx$$

$$= \frac{1}{\gamma^5} \int_{-\infty}^{+\infty} (1+x^2)^{-3} dx$$

The reduction technique is used. It is so cool and it is used for so many different integrals.

$$= \frac{1}{\gamma^5} \int_{-\infty}^{+\infty} (1+x^2)^{-2} \frac{d}{dx} (\arctan(x)) dx$$

The integration by parts is used, so let $u = (1+x^2)^{-2}$ and $dv = \frac{d}{dx}(\arctan(x))dx$. Now we have:

$$= \frac{1}{\gamma^5} [(1+x^2)^{-2} \arctan(x) |_{-\infty}^{+\infty} - \int_{-\infty}^{+\infty} -4x(1+x^2)^{-3} \arctan(x) dx]$$

In the above equation, the first term goes to zero, and in the second term consider $u = \arctan(x)$ and $x = \tan(u)$ so $du = (1+x^2)^{-1} dx$. Now substituting everything in the above equation and we have:

$$= \frac{1}{\gamma^5} [4 \int_{-\frac{\pi}{2}}^{\frac{\pi}{2}} u \tan(u)(1+\tan^2(u))^{-2} du]$$

Let $u = x$

$$= \frac{1}{\gamma^5} [4 \int_{-\frac{\pi}{2}}^{\frac{\pi}{2}} x \tan(x)(1+\tan^2(x))^{-2} dx]$$

We know that $\tan(x) = \frac{\sin(x)}{\cos(x)}$ and then the above equation can be reduced to follows:

$$= \frac{1}{\gamma^5} [4 \int_{-\frac{\pi}{2}}^{\frac{\pi}{2}} x \sin(x) \cos^3(x) dx]$$

Now integration by parts is used again. Let $u = x$ and $dv = \sin(x)\cos^2(x)dx$

$$= \frac{1}{\gamma^5} [4x(-\frac{1}{4}) \cos^4(x) |_{-\frac{\pi}{2}}^{\frac{\pi}{2}} + \int_{-\frac{\pi}{2}}^{\frac{\pi}{2}} \cos^4(x) dx]$$

The reduction formula is used again. We have $\cos^4(x)$ and want to reduce it to $\cos^2(x)$ and by using the useful integral, we have:

$$= \frac{1}{\gamma^5} * \frac{3}{4} \int_{-\frac{\pi}{2}}^{\frac{\pi}{2}} \cos^2(x) dx$$

$$= \frac{1}{\gamma^5} * \frac{3\pi}{8}$$

Therefore, we evaluate the value of A1. using the same techniques A2 is evaluated as follows:

$$\underbrace{\int_{-\infty}^{+\infty} \frac{1}{[\gamma^2 + X^2]^2} dX}_{A2} \qquad \text{(A2)}$$

The variable change is used. Let $x = \frac{X}{\gamma}$, So

$$\underbrace{\int_{-\infty}^{+\infty} \frac{1}{[\gamma^2 + X^2]^2} dX}_{A2} = \frac{1}{\gamma^3} \int_{-\infty}^{+\infty} \frac{1}{(1 + X^2)^2} dX$$

By reduction formula, we have:

$$= \frac{1}{\gamma^3} [\int_{-\infty}^{+\infty} (1 + X^2)^{-1} \frac{d}{dx} (\arctan(x)) dX]$$

Now integration by parts is used, let $u = (1 + x^2)^{-1}$ and $dv = \frac{d}{dx}(\arctan(x))$ so we have:

$$= \frac{1}{\gamma^3} [\frac{\arctan(x)}{1 + x^2} |_{-\infty}^{+\infty} + 2\int_{-\infty}^{+\infty} \frac{x}{(1 + x^2)^2} \arctan(x) dx]$$

The first term goes to zero. Now u substation is used $u = \arctan(x)$ and then $du = (1 + x^2)^{-1} dx$ so plugging everything in the above formula and we have:

$$= \frac{1}{\gamma^3} * 2\int_{-\frac{\pi}{2}}^{+\frac{\pi}{2}} \frac{u \tan(u)}{1 + \tan^2(u)} du$$

Let $u = x$

$$= \frac{1}{\gamma^3} * 2\int_{-\frac{\pi}{2}}^{+\frac{\pi}{2}} \frac{x \tan(x)}{1 + \tan^2(x)} dx$$

We know that $\tan(x) = \frac{\sin(x)}{\cos(x)}$, So

$$= \frac{1}{\gamma^3} * 2\int_{-\frac{\pi}{2}}^{+\frac{\pi}{2}} x \sin(x) \cos(x) dx$$

Using integration by part again, let $u = x$ and $dv = \sin(x)\cos(x)dx$, So:

$$= \frac{1}{\gamma^3}[\underbrace{x\sin^2(x)|_{-\frac{\pi}{2}}^{+\frac{\pi}{2}}}_{\pi} - \int_{-\frac{\pi}{2}}^{+\frac{\pi}{2}} \sin^2(x)dx]$$

The left term is π and by and the right term is reduced by using the useful integral as follows:

$$= \frac{1}{\gamma^3}[\pi - (\underbrace{-\frac{1}{2}\sin(x)\cos(x)|_{-\frac{\pi}{2}}^{+\frac{\pi}{2}}}_{0} + \underbrace{\frac{1}{2}\int_{-\frac{\pi}{2}}^{+\frac{\pi}{2}} dx}_{\frac{\pi}{2}})]$$

Therefore, the value of A2 is:

$$= \frac{1}{\gamma^3} * \frac{\pi}{2}$$

In the next step, $I_{22}(\theta)$ is calculated. It is necessary to calculate relations 1, 2, and 3 to calculate $I_{22}(\theta)$ (Relations A1 and A2 have already been calculated, and the solution to relation A3 is given below).

$$I_{22}(\theta) = -E_\theta\left[\frac{4\gamma^2}{[\gamma^2+(x-x_0)^2]^2} - \frac{2}{\gamma^2+(x-x_0)^2} - \frac{1}{\gamma^2}\right]$$

$$= -\int_{-\infty}^{+\infty}\left[\frac{4\gamma^2}{[\gamma^2+(x-x_0)^2]^2} - \frac{2}{\gamma^2+(x-x_0)^2} - \frac{1}{\gamma^2}\right].\frac{\gamma}{\pi(\gamma^2+(x-x_0)^2)}dx$$

$$= \frac{-4\gamma^3}{\pi}\underbrace{\int_{-\infty}^{+\infty}\frac{1}{[\gamma^2+X^2]^3}dX}_{A1} + \frac{2\gamma}{\pi}\underbrace{\int_{-\infty}^{+\infty}\frac{1}{[\gamma^2+X^2]^2}dX}_{A2} + \frac{1}{\gamma\pi}\underbrace{\int_{-\infty}^{+\infty}\frac{1}{\gamma^2+X^2}dX}_{A3}$$

$$= \frac{-4\gamma^3}{\pi} * \frac{3\pi}{8\gamma^5} + \frac{2\gamma}{\pi} * \frac{\pi}{2\gamma^3} + \frac{1}{\gamma\pi} * \frac{\pi}{\gamma} = \frac{1}{2\gamma^2}$$

$$\underbrace{\int_{-\infty}^{+\infty}\frac{1}{\gamma^2+X^2}dX}_{A3} \qquad \text{(A3)}$$

We do a variable change. Let $x = \frac{X}{\gamma}$, So

$$\underbrace{\int_{-\infty}^{+\infty}\frac{1}{\gamma^2+X^2}dX}_{A3} = \frac{1}{\gamma}\int_{-\infty}^{+\infty}\frac{1}{1+x^2}dx$$

Therefore, the value of A3 is as follows:

$$= \frac{1}{\gamma} \arctan(x) |_{-\infty}^{+\infty} = \frac{\pi}{\gamma}$$

In addition, $I_{21}(\theta)$ and $I_{12}(\theta)$ calculations are provided as follows:

$$I_{21}(\theta) = I_{12}(\theta) = -E_{\theta}\left[\frac{-4\gamma(x - x_0)}{[\gamma^2 + (x - x_0)^2]^2} \right]$$

$$= -\int_{-\infty}^{+\infty} \frac{-4\gamma(x - x_0)}{[\gamma^2 + (x - x_0)^2]^2} \cdot \frac{\gamma}{\pi(\gamma^2 + (x - x_0)^2)} dx$$

$$= \frac{4\gamma^2}{\pi} \int_{-\infty}^{+\infty} \frac{x}{(\gamma^2 + x^2)^3} dx$$

$$= \frac{4\gamma^2}{\pi} * \frac{1}{\gamma^4} \int_{-\infty}^{+\infty} \frac{x}{(1 + x^2)^3} dx$$

$$= \frac{4\gamma^2}{\pi} * \frac{1}{\gamma^4} * \frac{-1}{4(1 + x^2)^2} |_{-\infty}^{+\infty} = 0$$
